# Analysis of the Effect of Electrode Materials on the Sensitivity of Quartz Crystal Microbalance

**DOI:** 10.3390/nano12060975

**Published:** 2022-03-16

**Authors:** Qiao Chen, Xianhe Huang, Yao Yao, Kunlei Mao

**Affiliations:** School of Automation Engineering, University of Electronic Science and Technology of China, No. 2006, Xiyuan Avenue, Chengdu 611731, China; qiaochen@std.uestc.edu.cn (Q.C.); kunleimao@std.uestc.edu.cn (K.M.)

**Keywords:** humidity sensor, quartz crystal microbalance (QCM), graphene oxide (GO), electrode material parameters, mass sensitivity

## Abstract

This paper investigated the effect of electrode materials on the performance of quartz crystal microbalance (QCM) sensors by means of theoretical calculation, experiment, and finite element analysis methods. First, we calculated the particle displacement amplitude and thus obtained the mass sensitivity function distribution of QCMs with gold, silver and aluminum electrodes, and found that the QCM with the gold electrode has the highest mass sensitivity at the center of the electrode. Then, we tested the humidity-sensing performance of QCMs with gold, silver, and aluminum electrodes using graphene oxide (GO) as the sensitive material, and found that the QCM with the gold electrode has higher humidity sensitivity. Finally, we used the finite element analysis software COMSOL Multiphysics to simulate the specific electrode material parameters that affect the sensitivity of the QCMs. The simulation results show that the density and Young’s modulus of the electrode material parameters mainly affect the sensitivity. The results of this paper are instructive for optimizing QCM sensor performance and improving the capability of QCM quantitative analysis.

## 1. Introduction

The quartz crystal microbalance (QCM), consisting of a vibrating quartz thin wafer sandwiched between two metal excitation electrodes, is a surface-sensitive analytical tool and a highly sensitive surface interface process analysis tool with nanogram-level sensitivity that can reflect in situ and in real time the mass changes on the surface of quartz wafers [1,2,3]. QCM’s features, including real-time monitoring, the characterization of membrane deposition, the detection of specific antigens and study of cell adhesion, and its simplicity of operation, digital output, simple back-end processing circuit and strong anti-interference ability compared to other analytical tools, mean that it is widely used in chemical, physical, material, biological and other fields [4,5,6].

It is noteworthy that researchers nowadays mainly focus on application-oriented studies of QCM, such as the vacuum film thickness monitor [7]; Noi et al. combined total internal reflection fluorescence microscopy with a quartz crystal microbalance to directly monitor the catabolic response of anthocyanins to individual amyloid β (Aβ) fibers [2]; Yao et al. combined QCM and moisture-sensitive materials to make a QCM humidity sensor [8,9,10]; and Kartanas et al. combined liquid chromatography with a QCM to achieve label-free quantitative analysis of protein mixtures [11]. The Sauerbrey equation is the basis for the study of QCM sensors [12],

(1) $$\Delta f=-C_{qcm}*\Delta m$$

where Δf, Δm, and $C_{qcm}$ represent the frequency shift, mass change, and mass sensitivity, respectively. Obviously, the mass sensitivity defined by the Sauerbrey equation ignores the influence of parameters such as electrode shape, thickness, size and material. The analysis of particle displacement amplitude for the electroded region and non-electroded region of the QCM by Josse et al. demonstrated that the mass sensitivity of the QCM is not a constant, but a Gaussian-type distribution [13]. Based on this, we have continued to investigate the effect of electrode shape, thickness and size on sensitivity [14,15,16,17]. 

**Figure 1:**
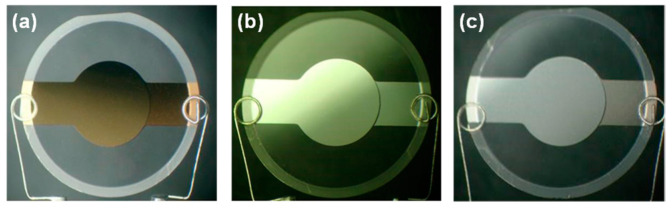
Photos of QCMs with (**a**) a gold electrode, (**b**) a silver electrode and (**c**) an aluminum Electrode.

The main electrode materials commonly used in QCM are gold, silver and aluminum, as shown in Figure 1. However, so far, researchers have generally agreed that there is no difference in their sensitivity and have not explained why a particular electrode material should be chosen [16].

In this paper, we first analyzed the particle displacement amplitude of QCMs with different electrode materials (densities) to compare their sensitivity. Then, QCM humidity sensors with different electrode materials were fabricated using graphene oxide (GO) as a sensitive material and their performances were compared. Finally, to further clarify exactly which material parameter affects the sensitivity of the QCM, simulation experiments were conducted using the finite element analysis software COMSOL Multiphysics.

## 2. Theory

According to previous works, the mass sensitivity of QCM exhibits a Gaussian distribution, which can be calculated by the following formula [13,18,19],

(2) $$S_{f}\left(r\right)=\frac{{\left|u\left(r\right)\right|}^{2}}{2\pi \int_{0}^{\infty }r{\left|u\left(r\right)\right|}^{2}dr}\cdot C_{f}$$

where $S_{f}\left(r\right)$ and $C_{f}$ are the mass sensitivity distribution and Sauerbrey sensitivity constant, respectively. r and u(r) are the distance from the electrode center and the particle displacement function, respectively. u(r) is obtained by the following Bessel equation [20].

(3) $$r^{2}\frac{\partial^{2}u}{\partial r^{2}}+r\frac{\partial u}{\partial r}+\frac{k_{i}^{2}r^{2}}{N}u=0$$

where *N* is determined by the material constants of quartz crystal. $k_{i}^{2}=\left(\omega^{2}-\omega_{i}^{2}\right)/c^{2}$, where i = *E*, *U* (*E* and *U* are the fully electroded region and non-electroded region, respectively). $\omega_{E}$ and $\omega_{U}$ are the cut-off frequency of the fully electroded region and the non-electroded region, respectively. *c* is the acoustic wave velocity in quartz crystal. 

**Figure 2:**
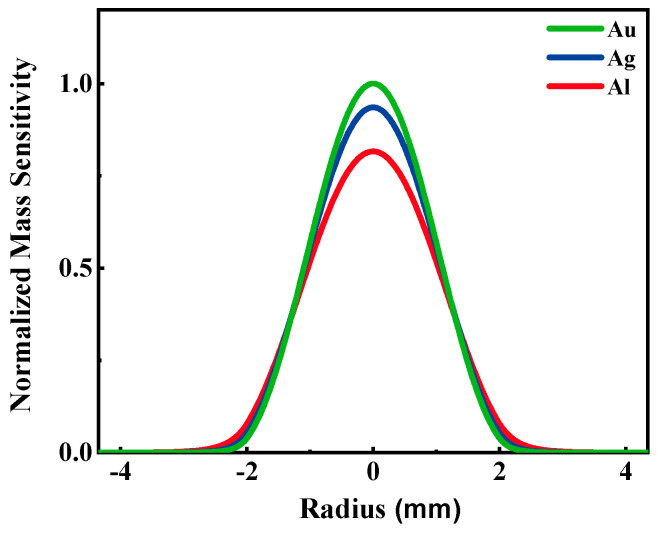
The normalized mass sensitivity distributions of QCMs with gold, silver, and aluminum electrodes.

The thickness and diameter of the wafer and electrodes used were 166 μm and 100 nm and 8.7 and 4 mm, respectively. The densities of gold, silver, and aluminum are 19.32, 10.5 and 2.7 g·cm^−3^, respectively. Bringing these values into the above equations, we obtained the normalized mass sensitivity functions of the QCMs with gold, silver, and aluminum electrodes, as shown in Figure 2. It can be seen that the gold electrode QCM has the highest mass sensitivity in the center of the electrode, followed by silver and the lowest for aluminum, which indicates that the electrode material affects the mass sensitivity of the QCM.

## 3. Experiment

**Figure 3:**
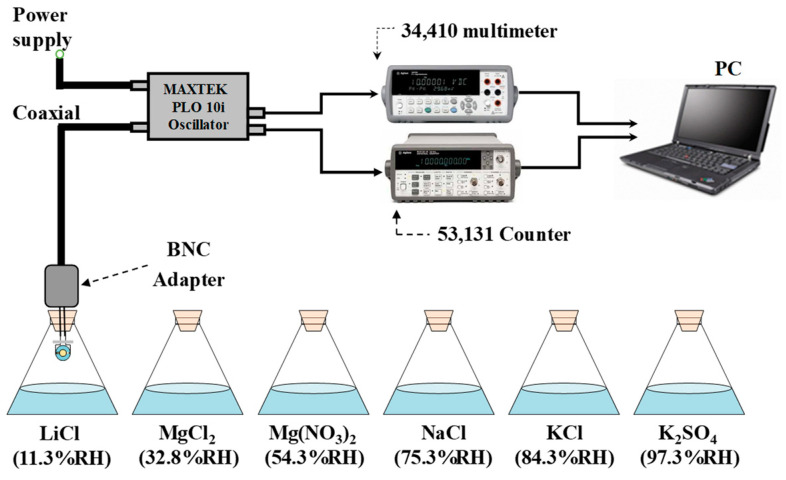
The schematic diagram of the humidity-sensitive experimental setup.

We investigated the effect of different electrode materials on the performance of QCM sensors using a humidity-sensing experiment. In general, a QCM humidity sensor mainly consists of a sensitive material and a QCM transducer [21]. Graphene oxide (GO) has strong hydrophilicity, a high mechanical modulus, and an ultra-high specific surface area, making it very suitable for fabrication as a humidity sensor [22]. In this paper, several QCM humidity sensors were fabricated using QCM transducers with gold, silver, and aluminum electrodes and GO as the humidity-sensing material. The schematic diagram of the humidity-sensitive experimental setup is shown in Figure 3. The phase-locked loop oscillator (PLO10i, Maxtek Inc., Santa Fe Springs, CA, USA) was connected to QCM sensors and provided the frequency signal. The frequency counter (53131A, Agilent Technologies, Santa Clara, CA, USA) and digital multimeter (34401A, Agilent) were used for recording the resonant frequency and voltage, respectively. Saturated LiCl, MgCl_2_, Mg(NO_3_)_2_, NaCl, KCl, and K_2_SO_4_ solutions at 25 °C were used to yield approximately 11.3%, 32.8%, 54.3%, 75.3%, 84.3%, and 97.3% RH levels, respectively. The experiment was conducted as a room temperature of 25 °C and an environmental humidity of 57% RH. The AT-cut, 10-MHz QCMs with gold, silver, and aluminum electrodes were purchased from Wintron Electronic Co., Ltd. (Zhengzhou, China). The diameters of the quartz plate and metal electrodes were 8.7 and 4 mm, respectively. The thicknesses of the quartz plate and metal electrodes were 166 μm and 100 nm, respectively. The GO solution of 2 mg/mL was obtained by means of a modified Hummers method [23]. A simple drop-coating method was used to deposit 3 μL GO solution on the QCMs with gold, silver, and aluminum electrodes, which were labeled Au-QCM, Ag-QCM, and Al-QCM, respectively. 

## 4. Results and Discussion

The frequencies of QCM sensors before and after attaching GO films were recorded in Table 1. The film formed by the same amount of GO solution causes different frequency shifts (Δf = $f_{1}-f_{2}$), which shows that the sensitivity of QCM sensors varies with different electrode materials.

**Figure 4:**
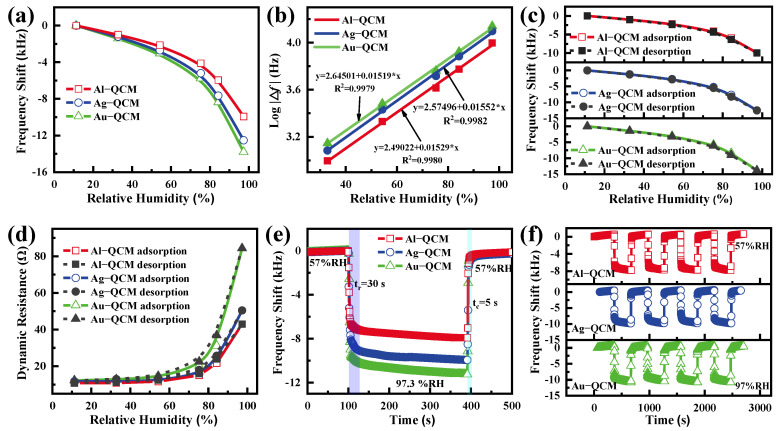
(**a**) The sensitivity, (**b**) logarithmic fitting curve of log|Δf| vs. RH, (**c**) humidity hysteresis, (**d**) dynamic resistance, (**e**) response/recovery time, and (**f**) repeatability of the Au-QCM, Ag-QCM, and Al-QCM.

The sensitivity is defined as the ratio of the frequency shift of the QCM sensor to the change in relative humidity, and the sensitivity of QCMs with different electrode materials in the relative humidity range of 11.3–97.3% is shown in Figure 4a. The maximum frequency changes for Au-QCM, Ag-QCM, and Al-QCM are about 13,800, 12,500, and 9950 Hz, respectively, and the humidity sensitivities are 160.5, 145.3, and 115.7 Hz/%RH, respectively. Additionally, it is clearly seen that the frequency variation of Au-QCM is greater than Ag-QCM and greater than AL-QCM at each humidity point, so we can conclude that the electrode material affects the sensitivity of QCM. Because the adsorption of water molecules by GO can be explained by the Langmuir adsorption model [24,25], as shown in Figure 4b, all QCM humidity sensors show an excellent logarithmic increase (log|Δf|) to the relative humidity level, and all regression coefficients ($R^{2}$) are around 0.998. 

Humidity hysteresis is defined as the ratio of the maximum frequency difference between the desorption and adsorption of water molecules to the frequency shift in the full humidity range. As shown in Figure 4c, the maximum frequency differences for Au-QCM, Ag-QCM, and Al-QCM are 630, 540, and 420 Hz, respectively. Therefore, the corresponding humidity hysteresis values are 4.56, 4.32, and 4.22% RH, respectively. This result shows that the electrode material has almost no effect on the humidity hysteresis. The Butterworth–Van Dyke (BVD) equivalent circuit model is often used to analyze the electro-acoustic behavior of QCM sensors. According to the literature [26], dynamic resistance can be used to effectively evaluate the stability of QCM humidity sensors. As shown in Figure 4d, there is 90 Ω > R_Au-QCM_ > R_Ag-QCM_ > R_Al-QCM_ during the adsorption or desorption of water molecules, which shows that all sensors have good stability in the full humidity range. Moreover, the stability of Al-QCM in the high humidity range is slightly higher than that of Ag-QCM and Au-QCM. The response/recovery time of a QCM humidity sensor is defined as the time it takes for the humidity sensor to reach 90% of the total frequency change. As shown in Figure 4e, the response/recovery times of all sensors are about 30/5 s between the environment humidity (57% RH) and 97.3% RH, indicating that the electrode materials have little effect on the response/recovery time. Finally, we tested the repeatability of all QCM humidity sensors by real-time recording the sensor’s response when it was repeated four times in the range of 57% RH–97.3% RH. As shown in Figure 4f, it can be seen that the frequency difference of all sensors has no obvious change, which shows the good repeatability of all QCM humidity sensors. 

**Figure 5:**
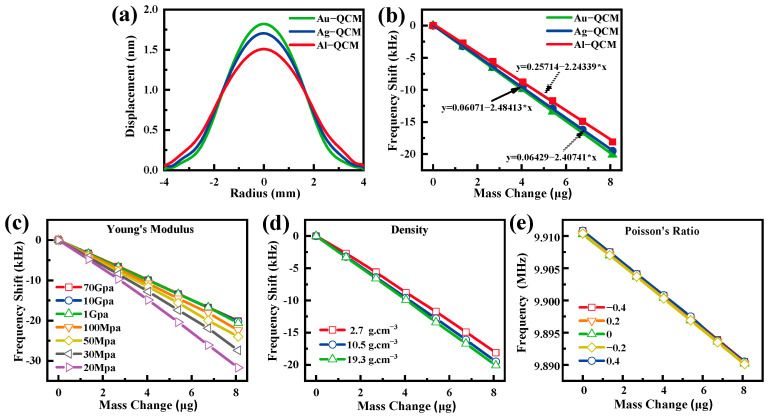
The simulation results of (**a**) the displacement and (**b**) sensitivity of Au-QCM, Ag-QCM, and Al-QCM. QCM’s simulation sensitivity at a different (**c**) Young’s modulus, (**d**) density, and (**e**) Poisson’s ratio.

The finite element analysis (FEM) method has been applied in many scientific computing fields due to its high calculation accuracy and wide application range [9]. In this article, we used the finite element analysis software COMSOL Multiphysics to analyze which electrode material parameter affects the sensitivity of the QCM sensor. The diameters and thickness of the quartz wafer and metal electrode were 8.7 mm, 166 μm, 4 mm, and 100 nm, respectively. First, we simulated the particle displacement amplitude of the QCM when the electrode materials were gold (19.3 g·cm^−3^), silver (10.5 g·cm^−3^), and aluminum (2.7 g·cm^−3^). As shown in Figure 5a, it can be seen that the displacement of Au-QCM is higher than that of Ag-QCM, and the displacement of Ag-QCM is higher than that of Al-QCM. According to the literature [13], the sensitivity of QCM is positively correlated with displacement. In addition, we also calculated the frequency change of QCM when the diameter of the additional mass was 3 mm. As shown in Figure 5b, it can be seen that the sensitivity of Au-QCM is higher than that of Ag-QCM, and the sensitivity of Ag-QCM is higher than that of Al-QCM, which is also consistent with our experimental results. Then, we used the controlled change method to study the influence of electrode material parameters (Young’s modulus, density, Poisson’s ratio) on the sensitivity of QCM. The relationship between the Young’s modulus of the electrode material and the QCM sensitivity is shown in Figure 5c. It can be seen that the sensitivity of the QCM sensor increases with the decrease in the Young’s modulus of electrode materials. When the Young’s modulus is greater than 1 GPa, continuing to increase the Young’s modulus will not affect the sensitivity. The Young’s modulus of gold, silver, and aluminum is far greater than 1 GPa. Therefore, we believe that the Young’s modulus does affect the sensitivity of Au-QCM, Ag-QCM, and Al-QCM. Then, we simulated the sensitivity at a density of 19.3, 10.5, and 2.7 g·cm^−3^. As shown in Figure 5d, it can be seen that the sensitivity of these three curves is almost the same as that of Au-QCM, Ag-QCM, and Al-QCM. This shows that the sensitivity of QCM of different materials is mainly affected by density. Finally, Figure 5e shows the relationship between Poisson’s ratio and QCM sensitivity. Several curves are almost coincident, indicating that Poisson’s ratio hardly affects the operating frequency and sensitivity of the QCM sensor. Therefore, researchers can choose to optimize the Young’s modulus of the electrode material or select a denser electrode material to further improve the sensitivity of the QCM sensor.

## 5. Conclusions

In this paper, we firstly calculated the particle displacement amplitude and thus obtained the mass sensitivity function distribution of QCMs with gold, silver and aluminum electrodes Then, GO was used as the sensitive material to test the humidity-sensing performance of QCMs with gold, silver, and aluminum electrode, and it was found that QCM with gold electrode material has higher sensitivity. Finally, we used the finite element analysis software COMSOL Multiphysics to simulate the specific electrode material parameters that affect the sensitivity of QCM. The simulation results show that the density and Young’s modulus of the electrode material mainly affect the sensitivity. The results have guiding significance for optimizing the performance of QCM-based sensors.

## Figures and Tables

**Table 1:** Frequency shifts before and after attaching GO films.

	$f_{1}\;(\mathrm{Hz})$	$f_{2}\;(\mathrm{Hz})$	Δf(Hz)
Au-QCM	9,960,680	9,943,400	17,280
Ag-QCM	9,996,550	9,980,120	16,430
Al-QCM	10,031,820	10,019,350	12,470
